# Significantly Positive Impact of Nonsteroidal Anti-inflammatory Drugs Combined With Osmoprotectant (Osmolytes) in Cancer Treatment

**DOI:** 10.7759/cureus.63529

**Published:** 2024-06-30

**Authors:** Mohd Basheeruddin, Sana Qausain

**Affiliations:** 1 Biochemistry, Jawaharlal Nehru Medical College, Datta Meghe Institute of Higher Education and Research, Wardha, IND; 2 Biomedical Sciences, Allied Health Sciences, Datta Meghe Institute of Higher Education and Research, Wardha, IND

**Keywords:** nsaids, inhibition of cox-2, cell death, cancer cells, osmolytes

## Abstract

Osmoprotectant osmolyte and nonsteroidal anti-inflammatory drug (NSAID) coadministration can work synergistically in cancer chemotherapy since most tumors are inflammatory and cancer cells experience osmotic stress. NSAIDs have been shown to inhibit cyclooxygenase (COX) enzymes, which in turn reduces prostaglandin synthesis and prevents inflammation. They also encourage cell death to prevent tumor growth and its spread to other tissues and prevent the construction of new blood vessels, which contributes to the growth of cancer. Taurine belongs to the class of osmolytes since it has been shown to stabilize macromolecular structures and maintain cellular osmotic balance when combined with betaine and glycine. When these drugs are taken together, as opposed to separately, the effectiveness of cancer treatment is increased by increasing cancer cell death and suppressing tumor growth.

Notable therapeutic benefits include the reduction of local inflammatory milieu by NSAIDs, which promotes tumor development, and the protection of surviving, normal cells and tissues from treatment-induced damage caused by cancer. By enhancing this synergy, side-effect risk can be decreased and treatment outcomes improved in terms of quality. Put another way, peptides can increase the therapeutic index of NSAIDs in cancer patients by preventing cell damage, which may lessen the gastrointestinal (GI), cardiovascular (CV), and renal side effects of the drug.

However, there are drawbacks because using NSAIDs for an extended period of time is linked to serious side effects that call for strict supervision. More research is required because the usefulness and significance of osmolytes in cancer therapy are still very unclear, if not fragmented. In addition, people who live in places with limited resources may find it difficult to afford the possible expenditures associated with osmolytes and selective cyclooxygenase-2 (COX-2) inhibitors.

Only the molecular mechanisms of the two drugs' interactions, the appropriate dosages for combination therapy, and clinical trials to validate the efficacy and safety of this dosage should be the focus of future research. The request is inviting because it presents hope for an extremely successful antiviral strategy; nevertheless, in order to implement this approach successfully, it is likely to be necessary to create affordable formulations and scalable solutions that do not necessitate excessive treatment regimen individualization. Due to their complementary capacities to demonstrate anti-inflammatory and cytoprotective effects, Akta and 5-aminosalicylic acid (5-ASA) administration may thus represent a significant advancement in the treatment of cancer.

## Introduction and background

Cancer treatment has traditionally relied on methods like chemotherapy and radiation, which, despite their efficacy, often cause significant side effects. This has driven the exploration of adjunctive treatments aimed at enhancing therapeutic outcomes while reducing adverse effects. Recent research highlights the potential of combining nonsteroidal anti-inflammatory drugs (NSAIDs) with osmoprotectants (osmolytes) as a novel approach in cancer therapy [1]. NSAIDs are widely recognized for their ability to alleviate pain and reduce inflammation. In cancer treatment, NSAIDs have demonstrated promise due to their capacity to inhibit cyclooxygenase (COX) enzymes, which play a critical role in inflammation, cell proliferation, and angiogenesis-key processes in cancer progression. By targeting these pathways, NSAIDs can potentially reduce tumor growth and metastasis [2].

Osmoprotectants, also known as osmolytes, are small organic molecules that help cells maintain osmotic balance and protect against stress-induced damage. In the context of cancer treatment, osmolytes such as taurine, betaine, and inositol can shield normal cells from the harmful effects of chemotherapy and radiation. They achieve this by stabilizing proteins and cellular structures, thereby enhancing cell survival and function under stress conditions [3].

Adjunctive treatments are promising, in addressing these hurdles, to augment the effectiveness of the existing treatment modalities for cancer without increasing the toxic effects of the interventions. The inadequately studied benefits of adjunctive therapies include the preservation of usually well-tolerated normal tissue while decreasing inflammation and/or enhancing the patient’s tolerance to chemotherapy and radiation, helping to maintain the dose intensity and scheduling schedule of the primary therapy, thus leading to improved treatment outcomes. Furthermore, these therapies would also appear to have the advantage over conventional cancer treatments in that they could reduce some of the long-term consequences for health that accompany chemotherapy, radiation therapy, or surgery. In conclusion, the incorporation of complementary and alternative medicine (CAM) therapy into conventional cancer treatment model is a potentially effective approach to manage cancer-related symptoms to improve the efficacy of therapies and the quality of life among cancer patients and the global cost of cancer management [4].

The aim of this review is to convey a summary of the noteworthy benefits of using NSAIDs in conjunction with osmolytes as cancer treatments. Additionally, it will highlight newly suggested drug-osmolyte combination therapies that are presently under investigation at the research stage and have the potential to address the drawbacks of traditional therapy. Numerous methods of cancer therapy and treatment will be covered, together with their current state in the clinical setting, emphasizing their value as cutting-edge anticancer tactics [5].

## Review

Information on cancer treatment challenges

At some time in their life, one in three people will develop cancer, making it the most lethal disease in the developed world. Since most current cancer treatments are insufficiently effective to fully defend against this disease, finding a cure is akin to finding the Holy Grail. We can now better understand the intricate relationships between several cellular genes and regulatory genetic variables that result in the aberrant phenotypes thanks to the recent development of sophisticated genomic, proteomic, and bioinformatics techniques [6].

As an illness, cancer affects people throughout all stages of their lives. Throughout human history, it has shown up in human mummies and hominid fossils. It was initially documented by Greek and Egyptian physicians. Cancer tears through society and causes pain on a worldwide level, affecting people of all ages. As per the World Health Organization, cancer ranks second globally in terms of cause of death, accounting for one in six fatalities [7].

Causes of the Challenges in Cancer Therapy

Cancer stem cells (CSCs) are challenging to target: According to an increasing amount of research, malignant cells frequently develop from a single stem cell-like cell. The way that cancer is treated ought to be significantly changed by these results. On the premise that every somatic cell has the potential to become malignant, traditional cancer treatment is predicated. These methods don't work to prevent cancer over the long term since they aren't much targeted. As CSC research is still in its early stages, there are a few obstacles to be addressed before identifying and focusing on CSCs [8]. It's unclear, for instance, if a progenitor cancer cell can learn to replicate itself.

CSCs are resistant to anticancer medications with drug resistance: Normal stem cells must continually undergo the processes of self-renewal and differentiation throughout an individual's life; thus, they have evolved unique defense systems to ward off dangerous xenobiotic chemicals. For instance, stem cells often express high numbers of adenosine triphosphate (ATP)-binding cassette transporter proteins. This protein is in charge of the drug efflux in breast cancer and has previously been found in a number of stem cells. Acute myelogenous leukemia (AML) stem cell subpopulations, known as CD34+/CD- cells, were also shown to have high expression levels of the breast cancer resistance protein (BCRP-ABCG2 protein), which was initiated to be actively implicated in drug efflux [9]. CSCs have the ability to confer drug resistance through different mechanisms.

Challenges in Cancer Therapy

Heterogeneity, low response rate, adverse effect, recurrence, metastasis, and drug resistance: The way that cancer is being treated remains reductionist despite significant advancements. But using a single medication as a "magic bullet" to target a specific characteristic or route is unlikely to result in the cure of cancer. A viable therapeutic method for cancer treatment in the near future, similar to human immunodeficiency virus (HIV) treatment, may be drug combinations (NSAIDs) against many molecular alterations or disease hallmarks. Combining immunotherapies with other anticancer drugs, such as targeted drugs in oncogene-driven malignancies, or with one another (two check point inhibitors, or a check point inhibitor plus an immunostimulatory antibody) is another option [10].

Outline of NSAIDs and osmoprotectants (osmolytes)

With over 30 million users worldwide, NSAID medications, or NSAIDs, are one of the prescription drug types most often used. Every year, NSAID-related kidney problems affect more than 2.5 million Americans. Account of diseases brought on by NSAIDs, the renal becomes more and more dependent on prostaglandins (PGs) to maintain kidney blood flow and glomerular filtration rate (GFR) [11]. Ongoing use may have unfavorable side effects. Pain, irritation, and stiffness are commonly treated with one of the NSAIDs. The adverse effects of excessive medication dosages include acute hepatitis, cell damage, and permanent cell death. A significant (p < 0.001) change in the histology of kidney, liver, heart, lungs, and heart tissue was seen following medication administration. Research into the structural stability of proteins essential in normal organ function and the detrimental effects of NSAIDs is therefore imperative. Furthermore, a drug's interaction with proteins has a significant impact on how it moves through the body; thus, it's critical to comprehend their molecular interactions [12].

NSAIDs and Acute Tissue Failure

Analgesic nephropathy, a slowly progressing chronic kidney disease, is brought on by long-term use of formulations containing at least two analgesics. These formulations include phenacetin, aspirin, acetaminophen, or pyrazolones as well as centrally acting medications that induce dependence, such as codeine, barbiturates, and caffeine, among others. There have also been reports of renal, lung, and liver papillary necrosis caused by conventional NSAIDs, which is depicted in Table 1 such as naproxen, ibuprofen, tolmetin, indomethacin, and benoxaprofen, even though this condition is limited to those with analgesic nephropathy [13]. The expression of cyclooxygenase-2 (COX-2) in the macula dense is reduced by endogenous angiotensin II. On the other hand, significantly increase COX-2 expression and may worsen tissue dysfunction linked to NSAIDs [14].

**Table 1:** Important nonsteroidal anti-inflammatory drugs (NSAIDs) on high dose which leads to tissue dysfunction This table was created by the author

	NSAIDs	Tissues
1.	Diclofenac	Kidney
2.	Ibuprofen	Liver
3.	Aspirin	Liver, kidney
4.	Naproxen	Intestine
5.	Celecoxib	Heart
6.	Indomethacin	Liver, kidney, heart
7.	Etodolac	Liver, stomach

Osmolytes “The Chemical Chaperones”

To defend themselves against the damaging effects of extreme environmental circumstances as excessive salt, high temperature, and desiccation, living cells accumulate compensating solutes. It has proven possible to stabilize proteins and cells in vitro by the use of compensatory solute properties. Among the naturally occurring compounds are various methylamines, amino acids, and their derivatives. In addition to protecting intracellular proteins of particular organisms from denaturation stress, prior studies have indicated that these osmolytes may also be able to protect any protein from this type of stress. It is also known that these chemicals have negligible to no effect on the functionality of those macromolecules [15].

Classification of Osmolytes

Amino acid and methyl ammonium chemical families are the top two families of osmolytes based on structural similarities. Glycine is an example of an amino acid, and alanine (found in betaine and sarcosine) is one of its derivatives. The way they impact the functionality of proteins determines whether they are considered counteracting or compatible. Osmolytes that are compatible increase the denaturation resistance of proteins while having little to no effect on their natural function [16]. Examples of this family include glycine and β-alanine, which are derivatives of certain amino acids. The unique ability of the methylamine class of osmolytes to defend intracellular proteins from the destabilizing and inactivating impact of urea is thought to exist. Counteracting osmolytes are thought to affect protein activity in the opposite way as compatible osmolytes, whereas urea alters protein function. The main osmolyte-counteracting agent in the kidneys of mammals is called betaine [17].

Highlighting the Potential Benefits of Combining NSAIDs with Osmoprotectants in Cancer Treatment

The available literature contains a wealth of information about NSAIDs' ability to prevent cancer, which is depicted in Table 2. These medications have been linked to a lower cancer risk in a number of cancer types, including breast, prostate [18], colorectal, ovarian [19], and head and neck cancers [20]. Many of these studies are epidemiologic in character.

**Table 2:** Important nonsteroidal anti-inflammatory drugs (NSAIDs) and their beneficial effects on tissue cancer This table was created by the author

	NSAIDs	Tissue cancer cells
1.	Diclofenac	Melanoma, lymphoma, and prostate carcinoma
2.	Ibuprofen	Thyroid cancer
3.	Aspirin	Hepatocellular carcinoma
4.	Naproxen	Urinary bladder cancer
5.	Celecoxib	Colon cancer
6.	Indomethacin	Gastric cancer
7.	Etodolac	Colorectal cancer

**Figure 1:**
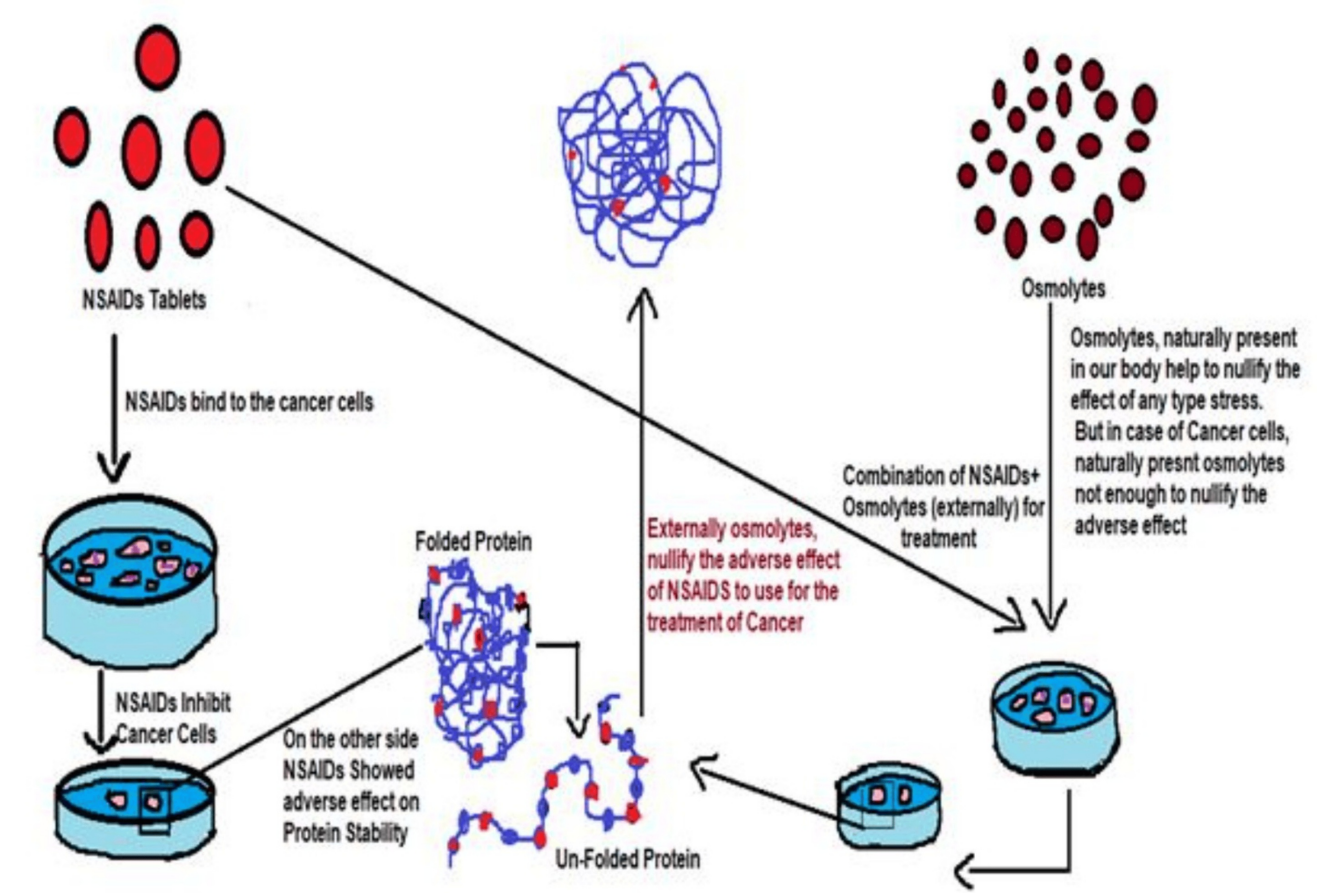
Schematic representation showing the effect of osmolytes with NSAID combination for the treatment of cancer NSAID: Nonsteroidal anti-inflammatory drug This image is the author's own creation

However, due to conflicting and contradictory research, the significance of NSAIDs in cancer prevention is yet unknown. Some research showed a decreased risk of cancer, but other studies showed no connection between NSAID use and cancer. For instance, non-aspirin NSAIDs were linked to no increased risk of ovarian or uterine cancer in a prospective research including around 20,000 women (aged 58-76) [21]. Protein and cell stability in vitro has been achieved through the use of compensatory properties. Nevertheless, the link between the use of NSAIDs and osmolytes in combination with cancer is complicated, which is depicted in Figure 1, and it is definitely oversimplified to assume that medications that reduce inflammation also prevent cancer.

Understanding cancer and inflammation

Inflammation and Cancer

Infections and inflammation have long been linked to cancer, and there are clear links between the emergence of precancerous lesions at different anatomic locations and the presence of inflammation. The risk of prostate cancer is around 14% higher in cases of prostatitis, 25% higher in cases of colon cancer due to ulcerative colitis, and 10-20 times higher in cases of pancreatic cancer. Accordingly, inflammation might supply the essential mutations as well as the ideal conditions for tumor growth. Inflammation is linked to the initiation, progression, and/or aggressiveness of many malignancies, according to a large body of research. Tumors exhibit characteristics of inflammatory cells as they grow [22]. For instance, many cancer cells have the receptors for cytokines and chemokine.

Cancer Therapy by Targeting Inflammation

Due to its many pro-tumor effects, inflammation is increasingly a focus for cancer prevention and treatment. The most often evaluated anticancer anti-inflammatory target is COX-2 and prostaglandin-endoperoxide synthase 2 (PTGS2), despite the fact that a number of other targets, including nuclear factor kappa B (NF-kB), cytokines/cytokine receptors, chemokines/chemokine receptors, fibroblast growth factor/receptor (FGF/FGFR), and vascular endothelial growth factor (VEGF), have also been investigated [23]. It was shown more than 20 years ago that NSAIDs prevent colon cancer. Early research concentrated on a range of broad-spectrum NSAIDs that nonspecifically inhibit cyclooxygenase-1 (COX-1) and COX-2; however, more recent research has looked at COX-2-specific drugs, like celecoxib. Many anti-inflammatory medications, including NSAIDs, might alter the tumor or the surrounding environment of the tumor, which can decrease migration, promote apoptosis, or make the tumor more sensitive to other therapies [24]. For these reasons, anti-inflammatory drugs continue to hold great promise in the fight against cancer. The next sections of this review will focus on the current state of the art, the underlying theories, and the future of anti-inflammatory medication therapy for cancer.

The Present Situation With Anti-inflammatory Drugs for Cancer

Diabetes, heart disease, psoriasis, arthritis, and cancer are among the chronic disorders that are significantly impacted by deregulated inflammation. Consequently, a wide range of anti-inflammatory drugs have FDA approval for these purposes. A number of these medications also have extra qualities like antiemetic and antithrombotic, proapoptotic, antiangiogenic, and antiproliferative properties, which makes them appealing options for cancer treatment or prevention, even if their original purpose was to reduce or avoid inflammation [25].

NSAIDs: The use of different NSAIDs, depicted in Table 3, such as medications like aspirin, is linked with a lower incidence of lung, colorectal, and breast cancers, according to epidemiological statistics. Aspirin and indomethacin users did not have a lower risk of malignancy during an extreme of 10 years of continuation, according to other studies, one of which included breast cancer patients. In spite of the fact that numerous clinical trials have not demonstrated any appreciable upgrading for enduring inhibition of various malignancies (studies with <10 years continuation), aspirin, diclofenac, and indomethacin use (300 mg/day for ≥5 years) can lower the enduring risk of colon adenomas after a 10-year latency period [26]. Another NSAID, sulindac, has also been demonstrated to cause the regression of pre-existing adenomas (39) and decrease the quantity of polyps and recurrences in familial adenomatous polyposis (FAP) patients. It has also been demonstrated that piroxicam and ibuprofen, two other NSAIDs, reduce the incidence of cancer [27].

**Table 3:** Important nonsteroidal anti-inflammatory drugs (NSAIDs) for stimulation and inhibition This table was created by the author

Nonsteroidal anti-inflammatory drugs (NSAIDs)	
1.	Diclofenac
2.	Aspirin
3.	Ibuprofen
4.	Sulindac
5.	Celecoxib
6.	Indomethacin
7.	Piroxicam

Specialized inhibitors of COX-2: NSAIDs are effective anti-inflammatory medications, but in an determination to create further dynamic complexes with less gastrointestinal (GI) damage, more recent medications have been developed to selectively block COX-2. Due to these results, the FDA expedited the approval of celecoxib in 1999 for use as an adjuvant therapy for FAP. Research has also suggested that celecoxib might be effective against malignancies other than ovarian cancer. Celecoxib and other NSAIDs are not shown to have any appreciable positive benefits on patients, according to numerous clinical trials; therefore, their use for cancer prevention and treatment is still debatable. Furthermore, there is less enthusiasm for the use of NSAIDs, particularly in the inhibition of malignancy, due to their adverse effects, which range from cardiotoxicity to moderate GI toxicity. The majority of COX-2-specific medicines have been taken off the market due to severe cardiotoxicity [28].

Corticosteroids: Corticosteroids have demonstrated anti-malignancy effectiveness when administered alone or in conjunction with chemotherapeutic medicines. They are most typically used to prevent or lessen the negative effects of radiation and chemotherapy. For instance, dietary administration of the glucocorticoid dexamethasone resulted in a greater than 60% reduction in the frequency of lung tumors in A/J mice exposed to tobacco smoke. Our research has shown that administering dexamethasone before to therapy can improve the outcomes of standard treatments for glioma in animal models as well as for lung, colon, and breast malignancies. Actually, coadministration of dexamethasone increased carboplatin and/or gemcitabine's effectiveness by 2-4 times [23].

NSAIDs in cancer treatment

NSAIDs Have Historically Been Used to Treat Cancer

Drug repurposing, the process of discovering new therapeutic effects for medications that have already received approval, is made possible by the polypharmacological profiles of pharmaceuticals, which let researchers find and highlight new therapeutic targets. Computational tools with systematic and reasonable methodologies to be applied beside various disease models may reveal new therapeutic functions for established medications in addition to medical observations or investigational data [29]. It is commonly known that under ideal circumstances, researchers must work on a novel chemical for 13-16 years before it is ready for market release. Preclinical research and clinical trials incur 2-3 billion dollars during the de novo drug discovery process. We attempted to assess significant in vitro and in vivo preclinical data about NSAIDs' anticancer effects in this section of the review. But in the history of this complex, fatal illness, the groundbreaking research of the German chemist Paul Ehrlich resulted in the successful application of chemicals for treatment [30]. Oncologists now have a variety of chemotherapy alternatives at their disposal.

Preclinical data from in vivo and in vitro experiments: Based on clinical data over the last 10 years, the use of NSAIDs, particularly aspirin and celecoxib, in the prevention and treatment of gastric and colorectal malignancies was reviewed in the previous section. The results of the studies conducted in the previous four years (2018-2021), both in vitro and in vivo, regarding the possible use of NSAIDs in the treatment and prevention of additional cancer types, were described in the current subsection with an emphasis on their mode of action. The anticancer effects of aspirin, celecoxib, nimesulide, and ibuprofen on lung, breast, hepatocarcinoma, pancreatic, and melanoma cancers were carefully evaluated in order to achieve this. Furthermore, the studies illustrating their modulatory roles in CSCs were included [19].

Benefits of aspirin against cancer: Li et al. [31] found that in HCC827 lung and MCF-7 breast cancer cell lines, aspirin caused apoptosis by upregulating the levels of cleaved poly-ADP ribose polymerase (PARP) and caspase-3. Aspirin treatment was observed to reduce the expression levels of CSC markers, CD44 and ALDH1A1, in lung cancer cell lines HCC827GR and HCC827OR [32]. Furthermore, Zhang et al.'s study [33] has shown that aspirin lowered the protein levels of programmed cell death-ligand 1 (PD-L1), a protein produced in tumor cells that inhibits antitumor immunity in human lung cancer cell lines A549 and H1299. Zhang et al.'s work used aldehyde dehydrogenase (ALDH) labeling in A549 lung, MDA-MB231 breast, and HepG2 hepatocellular carcinoma cell lines to examine alterations in the ALDH + subpopulation. All of these trials point to the possibility that NSAIDs, particularly when used in conjunction with licensed chemotherapeutic drugs, may be helpful in chemoprevention and cancer treatment. However, for their clinical applications as adjuvant therapy, controlled clinical trials with well-established outcomes have been desperately needed [33].

 *Mechanisms of Action of NSAIDs in Relation to Cancer*

Although the precise mechanisms underlying the promotion and maintenance of colon carcinogenesis remain unknown, research indicates that a complex interplay between genetic mutations and environmental carcinogens promotes the selective growth of transformed cells, ultimately resulting in the development of colonic dysplasia and cancer [34]. Colonoscopy screening, which looks for and removes precancerous lesions, or polyps, is the gold standard of CRC preventative methods, despite being an intrusive treatment that decreases compliance and involvement of CRC high-risk people in screening programs.

NSAIDs' action mechanisms in CRC chemoprevention: NSAIDs and aspirin control the transcription of genes and the synthesis of proteins in several molecules involved in inflammatory and neoplastic pathways. It is often possible to discern between these effects by looking at how aspirin and NSAIDs decrease the expression/activity of COX and downstream signals, which are critical for CRC cell proliferation, survival, and dissemination. Consequently, in the paragraphs that follow, the antineoplastic effects of NSAIDs and mesalazine will be discussed taking into account both COX-dependent and COX-independent pathways.

**Figure 2:**
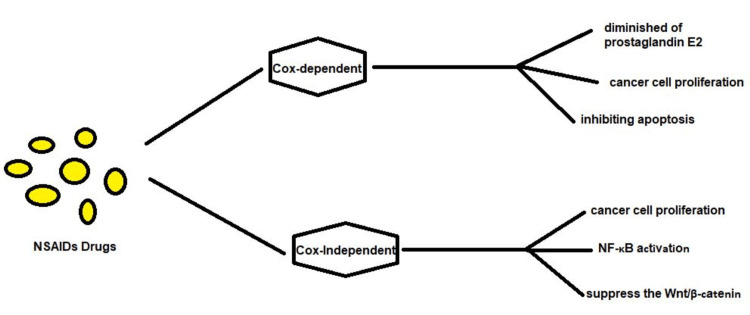
Overview of the NSAID-related Cox-dependent and independent mechanisms. This image is the author's own creation

COX-dependent mechanisms: In the context of cancer, NSAIDs act majored on COX-dependent pathways in a fashion that mostly involves the inhibition of the COX-2 enzyme that is commonly overexpressed in tumors. This inhibition results in diminished prostaglandin E2 synthesis, which is depicted in Figure 2, which is a prospective molecule in cancer advance. Prostaglandin E2 (PGE2) promotes tumor formation by promoting neoangiogenesis resulting from increased VEGF synthesis, stimulating cancer cell proliferation through the activation of signaling pathways that include ERK/MAPK and PI3K/AKT and inhibiting apoptosis, through the upregulation of antiapoptotic proteins while downregulating proapoptotic proteins. Interleukin-10 and PGE2, on the other hand, act directly on the tumor tissues and suppress the immune response to the tumors by limiting the activity of natural killers and cytotoxic T lymphocytes [35]. Through this interaction, the anti-inflammatory activity of NSAIDs decreases PGE2 concentration, which is crucial for the regulation of various pro-tumorigenic processes, thus rendering anticancer properties

COX-independent mechanisms: In the context of cancer, NSAIDs act through COX-independent mechanisms, by affecting some important signaling pathways, which are implicated in cancer cell proliferation as depicted in Figure 2, and survival. One of the signaling cascades affected by NSAIDs is the NF-κB signalingthat targets inflammation, cancer, and growth; NSAIDs prevent NF-κB activation thus decreasing the translation and expression of genes that favor cell growth and supply genes necessary for its progression. Furthermore, NSAIDs suppress the Wnt/β-cateninsignaling which is essential for cell proliferation and differentiation for cancers and, therefore, stop tumor formation [36]. This blockade is important because NSAIDs also cause cell death directly through pathways not related to COX inhibition, for example, through stimulating caspases and through causing the release of cytochrome c from the mitochondria. In addition, NSAIDs have been observed to diminish matrix metalloproteinases' (MMPs) mRNA which in turn minimizes the invasion of cancer cells and formation of metastases. These combined effects on COX-independent pathways largely account for the anticancer effects of NSAIDs, and this makes them important in the prevention and treatment of cancer [37].

Osmolyte actions: Compatible solutes, or osmolytes, for example, amino acids (such as proline), polyols (that include glycerol), and methylamines (featuring TMAO) are being indispensable for stabilizing proteins and various cellular structures in adverse conditions that may comprise high ion concentration, dehydration, or exposure to high/low temperatures [38]**. **They solely play their role in stabilizing the proteins mainly by preferential exclusion, by also favoring hydrophobic interactions besides directly interacting with the protein to facilitate the folding and maintain the native conformation. Furthermore, osmolytes prevent the disruption of the lipid bilayer, thereby decreasing the changes in membrane permeability, and strengthen the cytoskeleton elements by increasing the stability of actin and microtubule filaments. In this regard, osmolytes are involved in the regulation of the cellular stress responses, maintaining the functional integrity and structure of the cells, by modulating stress response pathways such as heat-shock response , osmotic stress response, etc.[39].

Overview and mode of action of NSAIDS: Analgesic, antipyretic, and NSAIDs are a broad family of pharmaceuticals. Salicylates, like aspirin; propionic acid derivatives, like ibuprofen and naproxen sodium; and para-aminophenols, like acetaminophen, are the three types of NSAIDs that the US Food and Drug Administration (FDA) has approved for over-the-counter (OTC) analgesic use. Ibuprofen and naproxen are recognized as traditional NSAIDs, whereas acetaminophen is not. Particularly in the high-peroxide environment of osteoarthritis (OA)-affected joints, paracetamol appears to have only weak anti-inflammatory effects and inadequate inhibitory effectiveness against COX [40]. 

Discuss Potential Benefits and Limitations of NSAID Immunotherapy

Benefits of NSAIDs for pain relief: NSAIDs, in contrast to opioids, have a maximum dosage beyond which there is no more pain relief, albeit larger doses do have anti-inflammatory benefits. In clinical trials with people with arthritis, non-aspirin NSAIDs are comparable to aspirin in reducing pain, joint swelling, and duration of morning stiffness while also improving strength and mobility. Individual variations in the level of inflammation and nociception sensitization susceptibility may be partly responsible for the observed discrepancies in pain intensity between individuals with radiographically limited disease and those with more severe joint degeneration and minimal discomfort [41]. Ibuprofen and naproxen are two nonselective NSAIDs that have OTC and prescription dosage alternatives. Large-scale clinical trials have evaluated the safety and effectiveness of ibuprofen and naproxen OTC dosages [42].

NSAID Risks

**Figure 3:**
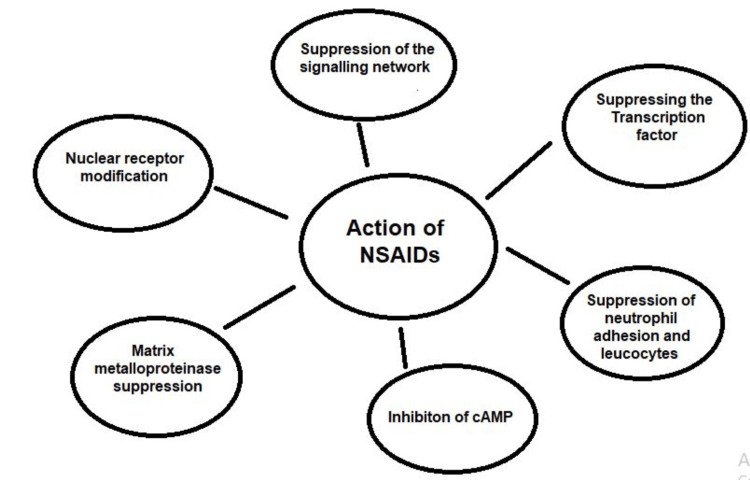
Diagrammatic representation showing the risk of NSAIDs NSAIDs: Nonsteroidal anti-inflammatory drugs This image was created by the author

GI effects: Heartburn, nausea, and dyspepsia are just a few of the GI adverse symptoms that have long been linked to NSAID use. According to endoscopic research, up to 30% of those who regularly take nonselective NSAIDs run the risk as depicted in Figure 3, developing duodenal or stomach ulcers. Patients receiving large dosages of NSAIDs, receiving treatment for prolonged periods of time, and using numerous NSAIDs concurrently are more likely to experience GI problems than those people who are older than 60, have had ulcers or bleeding problems in the past, or use aspirin, corticosteroids, or anticoagulants at the same time who are also at higher risk. Since 1992, GI issues and death linked to these medications have decreased with the use of combination therapy using proton-pump inhibitors and lower dosages of NSAIDs [43].

Cardiovascular (CV) effects: A number of large clinical trials have connected the COX-2-specific inhibitors to an increased risk of CV damage. Based on the Vioxx Gastrointestinal Outcomes Research (VIGOUR) trial, patients with rheumatoid arthritis treated with the COX-2-specific inhibitor rofecoxib had a fivefold increased risk of myocardial infarction compared to those treated with naproxen [44]. In a research called Adenoma Prevention with Celecoxib (APC), 22,035 individuals were given a placebo and two different dosages of celecoxib (200 mg and 400 mg twice day). The results showed a two- to threefold increase in relative risk of major CV events with celecoxib over placebo after a mean of 33 months. However, as compared to a placebo, 1561 participants in the Prevention of Colorectal Sporadic Adenomatous Polyps (PreSAP) trial did not show an increased CV risk when using celecoxib. Conversely, it was found that the CV safety of nonselective NSAIDs other than naproxen (mostly diclofenac and ibuprofen) was comparable to that of NSAIDs specific to COX-2. Additionally, compared to placebo, ibuprofen and diclofenac showed an increased risk of CV events; however, observational studies and indirect analyses of randomized trials demonstrated that the risk of CV events from naproxen was neutral [45].

Osmoprotectants in cancer therapy

Introduction to Osmoprotectants and Their Role in Cellular Protection

Osmoadaptation and its strategies: Variations in the external osmolality drive water fluxes along the osmotic gradient. Bacteria may swell and ultimately undergo lysis if they are subjected to an abrupt decrease in the ambient salinity, often known as an osmotic down shock [33]. Mechanosensitive ion channels are also found in eukaryotic membranes, such as those of plastids and fission yeasts, although little is known about their physiological function. Recent studies have shown that in response to osmotic stress, all other investigated organisms alter their cytoplasmic osmolality by manufacturing or absorbing specific osmolytes referred to as compatible solutes. The phrase suggests that these solutes accumulate without interfering with cellular macromolecules that are responsible for vital cellular functions, even when these solutes are found in significant amounts in the cytoplasm [46].

The stability of proteins affected by osmolytes in vivo: The stability of proteins inside the cells depends on osmolytes. The stability caused by osmolytes accelerates the pace at which proteins fold as depicted in Table 4. Protein folding in vivo, where the cell environment greatly influences folding, is a topic of great interest at the moment [38]. When animal cells are cultivated in the presence of either glycerol or deuterated water, they can withstand extreme heat-shock treatments that would typically be lethal to the cells. Osmosensitive transporters in the kidney indicate that within 3-6 hours of hyperosmotic exposure, Kaposi's sarcoma-associated herpesvirus (KSHV) produces the messenger RNA for the betaine transporter, which leads to intracellular accumulation of betaine. Releasing betaine occurs as the surrounding osmolality decreases. Betaine has the ability to mitigate the effects of osmolality on prostanoid production [38]**.**

**Table 4:** Important osmolytes which stabilize proteins This table was created by the author

Classification of Osmolytes					
Methylamines	Proteins	Polyols	Proteins	Amino acid derivatives	Proteins
Trimethylamine N-oxide (TMAO)	RNase T1, myoglobin	Sucrose	Trypsin and protein L	Proline	Phosphorylase b, myoglobin, creatine kinase
Betaine	RNase-A, lysozyme, and trypsinogen	Trehalose	Prion protein and mushroom tyrosinase	Glycine	Ribonuclease A, creatine kinase
Choline	Trastuzumab	Glycerol	Insulin	Ectoine	Trypsinogen, lysozyme
		Mannitol	Plasma protein and MAbs	Taurine	Ribonuclease A, lactate dehydrogenase
		Sorbitol	Gonadotropin, gamma-globulin	Serine	RNase-A, BSA
		Myo-Inositol	Human thyrocytes	Alanine	Lactalbumin
				Sarcosine	Ribonuclease A, stem bromelain

Osmolytes modulate protein stability: It is said that osmolytes evolved to regulate osmotic pressure and shield proteins from damaging external stimuli. The primary location of interaction with organic osmolytes is usually the peptide backbone. For almost a century, it has been known that urea denaturates proteins. But it wasn't until the 1960s that the denaturation of proteins by urea was actually established. Subsequently, there was a lot of interest in the opposite effect of stabilizing proteins by adding small amounts of organic osmolytes that exist naturally [47].

Protein stabilization by osmolytes-the mechanism: It has long been known that certain substances cause proteins in aqueous solutions to retain their original shape. To better understand this mechanism, studies of preferred interactions with components in a water glycerol solvent solution were conducted using six different proteins. They showed that all six of the proteins used had their preferred hydration promoted by the water-glycerol solvent combination [48] . A protein's chemical potential, or its activity coefficient, is enhanced by an increase in glycerol content, according to preferential binding data. Since glycerol repels nonpolar molecules and interacts positively with water, its presence in aqueous environments should increase the hydrophobicity of the protein.

Osmophobic effect: Bolen et al. have done research on osmolytes and related compounds in an effort to clarify the process of protein stabilization. Additionally, some of their research showed that these osmolytes tend to destabilize proteins at all doses. According to Bakasov and Bolen's research, urea, a denaturant, and trimethylamine N-oxide (TMAO), a potent stabilizer, are both required whether or not the proteins were generated in the presence of osmolytes [49]. Their findings indicate that both urea and TMAO have favorable side chain interactions overall and that both should denature proteins because urea is not any more effective than TMAO at solubilizing hydrophobic side chains. According to Bakasov and Bolen's research, urea, a denaturant, and TMAO, a potent stabilizer, are both required whether or not the proteins were generated in the presence of osmolytes.

Mechanisms of Action of Osmoprotectants in Relation to Cancer

Mechanism of osmolyte-induced protein stabilization: In the equilibrium protein folding reaction, protecting osmolytes push the equilibrium toward D while denaturing osmolytes push the equilibrium toward N. Currently, no comprehensive molecular theory can explain how osmolytes interact with proteins to change thermodynamic equilibrium. The strongest evidence, however, comes from Arakawa and colleagues' work, which demonstrates that the primary mechanism causing osmolyte-induced protein stabilization is the preferential exclusion of stabilizing osmolytes from the immediate vicinity of the protein domain. Using thermodynamic studies to assess how the chemical potentials of native and denatured proteins change when they are transferred to osmolyte solutions, Arakawa [50] also came to the same conclusion. It has also been demonstrated that these osmolytes have specific propensities to interact with the various protein surface groups, hence affecting the thermodynamic transition between protein states that reveal different kinds and amounts of surface area.

Clinical trials and case studies

Review Relevant Clinical Trials and Case Studies Demonstrating the Benefits of This Combination Therapy

When compared to isotonic controls (aspirin absent), Madin-Darby canine kidney (MDCK) cells that had previously acclimated to a hypertonic (500 mM) growth media for 24 hours displayed an over 10-fold elevation of betaine/γ-aminobutyric acid transporter 1 (BGT1) transport activity. In this first investigation, dose-dependent suppression of BGT1 transport was seen when aspirin (range: 0.1-1.0 mM) was added to the hypertonic medium and left for a further 24 hours. The inhibitory impact at 1.0 mM is comparable to the aspirin serum level (1-2 mM) that is useful in the treatment of rheumatoid arthritis [51]. NS-398 was employed at a final dose of 0.01 mM, which has been demonstrated to suppress PGE2 synthesis in the inner medullary collecting duct-3 (IMCD3) cells while having no effect on COX-1. Similar to BGT1 transport, the medicines were inert in isotonic circumstances, indicating that hypertonicity could make cells more susceptible to the effects of medications on these transport systems.

Results of clinical trial: This is the first account of how NSAIDs and analgesics impede renal osmolyte transport, with consistent results observed in two distinct lines of renal medullary cells. It is possible to rule out apoptosis and nonspecific toxicity, but the precise processes remain unknown. Acetylsalicylic acid, the OTC version of aspirin that is evaluated here in vitro, is quickly changed to salicylic acid after consumption. A COX-2 specific inhibitor was able to mimic the effects of aspirin, indicating that aspirin may function by inhibiting COX-2 and potentially depleting prostaglandins, which are crucial for sustaining the body's ability to adapt to hypertonic stress. Additional research is required to validate this.

Potential side effects and precautions

Beneficial and Harmful Effects of Intracellular Organic Osmolytes

Additionally covered are some pathophysiological effects of these chemicals' improper buildup. Many different types of bacteria, plants, and animals have been shown to have an effective defense against hypertonicity during water stress in the form of suitable organic osmolytes. There are three osmolyte groups identified: (1) polyhydric alcohols, (2) free amino acids and their byproducts, and (3) mixtures of urea and methylamines. Clark laid the theoretical foundation for comprehending this phenomenon by examining the potential uses of the high concentration of amino acids found in the cells of some euryhaline animals. Wyn Jones independently presented a similar theory for plants around the same period. Clark discovered that the cations and anions in the Hofmeister or lyotropic series that support "native" macromolecular structure are structurally similar to the neutral and acidic amino acids that are being studied [52].

Side Effects Analysis on Tissues

Prominently, they are used for the treatment of pain, inflammation, and fever; however, it carries with it considerable side effects; some of which are on the GI tract, CV, and renal systems.

GI issues: These adverse effects include GI ones and are considered the most frequent and serious consequences of taking NSAIDs. But since they are nonselective, they will block COX-1 which is involved in the generation of prostaglandin that has a cytoprotective effect in the gastric mucosa. This inhibition may cause a decrease in the blood flow to the mucosa, diminished secretion of mucus and bicarbonate in the gastric juice, and increased secretion of gastric acid, which ultimately leads to mucosal injury[53]. Gentleman nearing or already in the 60-year age range are at an increased risk of peptic ulceration, GI hemorrhage, or perforation on long-term NSAID use as shown by clinical data. For instance, a study revealed that 1,095 cases [54] of serious GI complications were estimated to occur among one-year continual NSAID users, with increased risk recorded among those in the higher dosage and longer usage.

CV issues: CV side effects are more common with the "coxibs"; however, they are also seen with other "conventional" NSAIDs. The use of COX-2 selective NSAIDs decreases the synthesis of prostacyclin which is a vasodilator with activities on platelet aggregation that can be associated with thrombotic events [55]. Epidemiological studies involving interacting with patients and case-control or cohort study demonstrate that coxib and high-threating nonselective NSAIDs such as diclofenac increase the risk of myocardial infarction, stroke, or congestive heart failure. For example, a meta-analysis suggested that patient users of high-dose diclofenac or coxibs were at an increased risk of developing CV diseases as much as users of rofecoxib which was pulled off the market due to CV risks [40] .

Renal issues: The undesired effects NSAIDs have on the kidney can be attributed to alteration of renal blood flow. Through blocking of COX enzymes in the kidneys, NSAIDs impair a renal component that plays a critical role in maintaining renal blood flow particularly in conditions accompanied by renally reduced renal blood flow [11]. Within this scenario, one can develop AKI, deranged electrolyte levels, as well as worsening of their CKD status. It has been suggested that NSAID use in clinical conditions was associated with significantly increased risk of renal dysfunction, particularly in those patients with previous histories of renal disease or those concurrently receiving other nephrotoxic drugs [56]. A cohort study of the risk exposed that the AKI hospitalizations among NSAID users were greater than among nonusers, thus indicating the huge renal dangers associated with these medicines.

Mitigation Strategies

Dosage optimization: To minimize these side effects, the use of the drug should be done most effectively by checking on the proper dosage. By ensuring that one uses the minimum dose that one requires, minimally for the shortest possible period, one can effectively minimize the risk of experiencing undesired effects of the drug. Also, monitoring the frequencies of NSAID use and reassessing whether the NSAID use is still required is the proper practice.

Alternative drug formulations: Other possibilities like topical NSAIDs can ensure significant pain reduction and has minimal penetration into systemic circulation and therefore pose a lower threat of possessing adverse GI and CV side effects [40]. For instance, topical therapy like diclofenac gel has been proved effective to give relief to localized pain having better side effects than oral preparations.

Combination therapies: It is also worth mentioning that the combination with other drugs may reduce the side effects of NSAIDs as well. Preparations can be co-prescribed with proton-pump inhibitors (PPIs) or H2-receptor antagonists that help to reduce asphalt acid production, thus reducing the dangers of GI complications associated with NSAIDs. To reduce CV risks, it is appropriate to employ the below NSAID of low CV risk, such as naproxen, as well as aimed at discontinuation of high-dose regimens [57]. Thirdly, switch to using acetaminophen for pain management or applying nonpharmacological ones when NSAIDs must be employed, it is possible to reduce the risk.

Future Perspectives and Challenges

The current review article can be expanded in the ways listed below:

It was demonstrated that NSAIDS would not aggregate when exposed to heat when sub-micellar concentrations of osmolytes changed it into an α-helical shape. It would be beneficial to look into the NSAID-protein interaction in the presence of other group osmolytes in addition to screening for other additives. This would allow one to ascertain if the interaction occurs directly or through a bridge molecule. Additionally, research can be done on the osmolytes' potential to improve the conformational stability of other commercially significant proteins with NSAIDS.

The current finding opens the door to a deeper comprehension of alternative processes underlying the interaction between NSAIDS and proteins. It would also be interesting to examine the effects of NSAIDS in the presence of various osmolyte groups in vivo and in vitro as well as to calculate the urea-osmolyte ratio under these circumstances.

Since osmolytes are an essential component of our diet, research on the negative effects of NSAIDS on proteins both in vitro and in vivo were observed. But given the promising results of the current study, it would be worthwhile to look into how NSAIDs affect particular organ proteins both in vitro and in vivo.

Review: Summarize the Main Findings and Contributions of the Review

Regarding cancer treatment, there are no "magic bullets." Because of the complexity of this disease, a multifaceted, mechanistically tailored strategy is needed. The main focus of cancer therapy strategies has been to kill the tumor cell. Approaches to alter the host microenvironment present a fresh viewpoint on cancer treatment. One major challenge has been how to address inflammation associated with malignancy in cancer patients. Comprehending the diverse biochemical and signaling pathways implicated in the dynamics of tumor cells has furnished the requisite instruments for formulating medications that either enhance or impede these prospective targets.

Prostanoid production is restricted by the inhibition of the COX pathway by aspirin and NSAIDs. The fact that NSAIDs are an irreversible inhibitor of COX acetylation, as opposed to reversible inhibitors, is what matters. Previous research has shown that aspirin acetylation of COX-2 alters the catalytic domain, preventing the synthesis of PG. Despite this, the enzyme is still active and in COX-2-expressing cells can produce 15R-HETE from arachidonic acid, 18R-HEPE from eicosapentaenoic acid (EPA), and 17R-HDHA from docosahexaenoic acid (DHA). These are converted by leukocytes into lipoxins, resolvins, and protectins that are induced by aspirin. These aspirin-triggered specialized pro-resolving mediators (AT-SPMs) are powerful mediators that improve macrophage cleaning and prevent polymorphonuclear leukocyte (PMN) invasion. It's interesting to note that COX-1 does not significantly create aspirin-triggered epimers and that COXIBs and NSAIDs both inhibit COX-1 and COX-2. Consequently, SPMs generated by aspirin have the potential to cause cancer resolution, indicating their potential value in conjunction with chemotherapy. Conversely, optoprotectants, or osmolytes, counteract or neutralize the effects of NSAIDs in the treatment of cancer. There is still a lot of work to be done in this field.

## Conclusions

In cancer treatment, the use of osmoprotectants (osmolytes) in conjunction with NSAIDs has the potential to improve therapeutic outcomes. NSAIDs are well-known for their anti-inflammatory, analgesic, and antipyretic qualities. By preventing inflammation that promotes tumor growth, lowering the growth of cancer cells, and triggering apoptosis, NSAIDs can help treat cancer. However, GI and CV adverse effects frequently restrict their therapeutic efficiency. Small organic molecules called osmoprotectants, which aid cells in adjusting to osmotic stress, may lessen these adverse effects and improve the overall efficacy of NSAIDs in treating cancer. Osmolytes, such as trehalose, taurine, and betaine, lessen oxidative stress, stabilize proteins, and support cellular homeostasis. These characteristics can lessen NSAID toxicity, increase patient tolerance, and strengthen anticancer effectiveness.

An important conceptual and practical issue is presented by the current practice of utilizing NSAIDs as osmolytes and anticancer drugs in cancer treatment. Because of these factors, the therapeutic benefits of NSAIDs must be weighed against their side effects, which include GI, CV, and renal consequences. For this reason, it is crucial to obtain patient consent before using NSAIDs. Because selective COX-2 inhibitors and osmolytes are very expensive, accessibility issues arise because not everyone can afford these medications, particularly in developing nations. Similar to other technical breakthroughs, these therapies mustn't place an excessive financial strain on the patient. Additionally, many of these treatments have side effects that need to be carefully managed to improve patient safety and accessibility to these technologies.

According to studies, this combination therapy can increase the death of cancer cells, slow the growth of tumors, and increase survival rates. Together, NSAIDs and osmoprotectants target several cancer pathways, which offers a safer and more effective therapy approach. Furthermore, investigating the molecular processes that underlie their synergistic effects would offer more profound understanding of their therapeutic potential and facilitate the development of individualized treatment plans.

To validate these results and improve treatment regimens, more clinical trials are necessary. The amalgamation of NSAIDs and osmoprotectants is a hopeful novel approach in cancer treatment, deserving of further investigation and practical testing.
